# Genomic insights into multidrug resistance and virulence of methicillin-resistant *Staphylococcus pseudintermedius* from companion animal otitis

**DOI:** 10.3389/fvets.2026.1759838

**Published:** 2026-01-28

**Authors:** Juliana Menezes, Manuel Rodrigues, Tiago Pinto-Lima, Sara Isidoro, André Meneses, Adriana Belas

**Affiliations:** 1 Faculty of Veterinary Medicine, Lusófona University - Lisbon University Centre, Lisbon, Portugal; 2 I-MVET - Research in Veterinary Medicine, Faculty of Veterinary Medicine, Lusófona University - Lisbon University Centre, Lisbon, Portugal; 3 Department of Immuno-Physiology and Pharmacology-Institute of Biomedical Sciences Abel Salazar (ICBAS), UP, Porto, Portugal; 4 LAQV@REQUIMTE, University of Porto (UP), Porto, Portugal; 5 IPLUSO – Superior School of Health, Protection and Animal Welfare, Polytechnic Institute of Lusophony, Lisbon, Portugal; 6 CECAV - Animal and Veterinary Research Center, Faculty of Veterinary Medicine, Lusófona University - Lisbon University Centre, Lisbon, Portugal

**Keywords:** MDR, mecA gene, methicillin-resistant *Staphylococcus haemolyticus*, MRSP, One Health, ST551, whole-genome sequencing (WGS)

## Abstract

Staphylococci are major opportunistic pathogens of companion animals and an important reservoir of antimicrobial resistance genes with zoonotic relevance. Otitis externa is one of the most common conditions requiring antimicrobial therapy in veterinary practice, yet data integrating phenotypic and genomic analyses of staphylococcal isolates remain limited. This retrospective study aimed to characterize *Staphylococcus* spp. isolates recovered from cases of otitis externa in dogs and cats. *Staphylococcus* spp. strains (*n* = 76) recovered from otitis cases in dogs and cats, which were identified by 16S rRNA gene sequencing, were tested for antimicrobial susceptibility according to CLSI guidelines. Methicillin-resistant strains (*n* = 11) were further characterized by whole-genome sequencing (WGS) to determine sequence types, resistance determinants, and virulence-associated genes. *Staphylococcus pseudintermedius* was the predominant species identified. A high proportion of strains exhibited resistance to tetracyclines and β-lactams, and 39.5% were classified as multidrug-resistant (MDR). Methicillin-resistant strains carried *mecA* and predominantly belonged to the European methicillin-resistant *S. pseudintermedius* (MRSP) lineage ST551, alongside ST496, ST1786, ST1095, and three novel sequence types. Genomic analysis revealed a conserved virulence repertoire including leukocidins, biofilm-associated genes (*icaBDCA, sdrD*), lipoprotein maturation enzymes (*lgt, lspA*), and immune-modulatory exotoxins. Core SNP-based analysis showed that two strains from dogs originating from different owners differed by only two SNPs. The combination of phenotypic resistance and genomic virulence determinants observed in these strains highlights the clinical significance of *Staphylococcus* spp. in otitis externa and reinforces the need for prudent antimicrobial stewardship and robust infection-control measures in veterinary medicine. The study also illustrates how sustained genomic surveillance can generate insights that support both veterinary and public-health actions.

## 1 Introduction

Staphylococci are facultative anaerobic, opportunistic pathogens capable of causing a broad spectrum of infections due to their diverse virulence factors and remarkable ability to acquire antimicrobial resistance (1, 2). Many staphylococcal species colonize both humans and animals and can act as reservoirs of antimicrobial resistance genes. Some are able to cause opportunistic infections and may be transmitted between hosts, contributing to the spread of resistant lineages (3–5). Within this context, staphylococci are of relevance from a One Health perspective (4).

Among companion animals, the coagulase-positive species *Staphylococcus pseudintermedius, Staphylococcus schleiferi*, and *Staphylococcus aureus* are the most commonly isolated from clinical infections, particularly skin and soft tissue infections (SSTIs) and urinary tract infections (6, 7). Otitis externa is one of the most common reasons for veterinary consultations and often requires antimicrobial therapy (8). *Staphylococcus pseudintermedius* is consistently reported as the predominant bacterial pathogen in such infections, with methicillin-resistant *S. pseudintermedius* (MRSP) posing a therapeutic challenge due to its multidrug-resistant (MDR) nature (7, 9).

Despite the well-established importance of MRSP, studies combining antimicrobial susceptibility profiling with genomic characterization remain scarce, particularly in Portugal, limiting our understanding of their clinical relevance, epidemiology and zoonotic potential. Knowledge gaps persist regarding the prevalence of sequence types, and virulence genes—including those mediating biofilm formation, which play a critical role in chronicity and persistence of infections (10, 11).

The presence of *mecA* or *mecC* genes is one of the most clinically relevant features within staphylococcal species from companion animals (12, 13). These genes encode an alternative penicillin-binding protein that confers resistance to β-lactam antibiotics—classified by the World Health Organization (14) as medically important antimicrobials and first-line agents in both human and veterinary medicine. Consequently, methicillin-resistant staphylococci (MRS) pose substantial therapeutic challenges and raise concerns regarding zoonotic transmission and treatment failures (4).

In the context of rising antimicrobial resistance (AMR) and the increasing demand for evidence-based antimicrobial stewardship, continuous surveillance of staphylococci from companion animals is crucial. Comprehensive characterization of resistance phenotypes, genetic determinants, lineages, and virulence factors can inform rational antimicrobial use and mitigate the dissemination of multidrug-resistant pathogens across animal and human populations.

This study aimed to characterize *Staphylococcus* spp. isolates from cases of otitis externa in dogs and cats in Lisbon, Portugal, by assessing antimicrobial resistance in all isolates, and performing whole-genome sequencing (WGS) of methicillin-resistant strains to evaluate their genetic diversity and virulence genes, to access their potential zoonotic relevance.

## 2 Materials and methods

### 2.1 Ethics approval

Ethical approval for the use of clinical isolates was obtained from the Ethics and Animal Welfare Committee of the Faculty of Veterinary Medicine, Lusófona University (Lisbon, Portugal), under the approval number 16-2025. All procedures were conducted in accordance with national and institutional guidelines for the care and use of animals in research.

### 2.2 Isolate collection

In this retrospective study, a total of 76 staphylococcal isolates were included, obtained between 2023 and 2024 from ear swab samples of dogs (*n* = 56) and cats (*n* = 20) presenting clinical signs of otitis externa. The clinical ear swab samples were submitted to the Microbiology Laboratory of the Faculty of Veterinary Medicine, Lusófona University (Lisbon, Portugal), together with a brief submission form containing information such as the animal's age, sex, clinical description of the lesion site, and suspected course of disease. Infections were classified as community-acquired if they were present at the time of consultation or within 48 h after hospital admission, indicating that the animals were infected prior to any clinical intervention. *Staphylococcus* spp. were isolated and stored in Brain Heart Infusion broth (Biokar, France) supplemented with 20% glycerol (Sigma-Aldrich, Portugal) at −80 °C. Each isolate was analyzed individually, and in cases where multiple isolates were obtained from the same animal (i.e., different staphylococci isolated at the same time from the same specimen or at different time points), each isolate was included as a separate entry only when the species or antimicrobial resistance profile differed.

### 2.3 Staphylococcal species identification

Presumptive identification of *Staphylococcus* spp. clinical isolates was performed by subculturing preserved isolates on Columbia Agar supplemented with 5% sheep blood (Biogerm, Portugal), following standard microbiological procedures.

Species-level identification was confirmed by sequencing the 16S rRNA gene (15). Bacterial DNA was extracted using a heat lysis and centrifugation protocol (16), and the supernatant containing DNA was stored at −20 °C until use. Each PCR reaction contained 1 × Supreme NZYTaq II Green Master Mix (NZYTech, Lisbon, Portugal), 0.5 μM of each primer (16S-1: 5'GTGCCAGCAGCCGCGGTAA 3'; 16S-2: 5'AGACCCGGGAACGTATTCAC 3'), and template DNA. Amplification was performed on a Biometra Uno II thermal cycler (Biometra Tone Series, Analytik Jena, Germany). PCR products were run by 1.5% (w/v) agarose gel electrophoresis, stained with GreenSafe Premium (NZYTech, Portugal), and visualized under UV light using a UView™ Mini Transilluminator (Bio-Rad, France). PCR products were then purified using the NZYTech Gel Pure Kit (NZYTech, Portugal) and sequencing was performed by StabVida (Caparica, Portugal). The obtained sequences were compared to published DNA sequences using Basic Local Alignment Search Tool (BLAST) (http://blast.ncbi.nlm.nih.gov/).

### 2.4 Antimicrobial susceptibility testing

All confirmed *Staphylococcus* spp. isolates were tested by disk diffusion according to Clinical and Laboratory Standards Institute (CLSI) guidelines. A total of 21 antimicrobial agents (Oxoid, Hampshire, UK) were tested: amoxicillin/clavulanate (30 μg), ampicillin (10 μg), cefovecin (30 μg), cefoxitin (30 μg), cephalothin (30 μg), chloramphenicol (30 μg), clindamycin (2 μg), doxycycline (30 μg), enrofloxacin (5 μg), erythromycin (15 μg), florfenicol (30 μg), gentamicin (10 μg), levofloxacin (5 μg), minocycline (30 μg), penicillin G (10 U), oxacillin (1 μg), rifampicin (5 μg), tobramycin (10 μg), trimethoprim–sulfamethoxazole (25 μg), fusidic acid (10 μg), and amikacin (30 μg). The D-zone test was performed to detect inducible clindamycin resistance. Presumptive β-lactamase production was inferred by observation of the inhibition zone edge around penicillin disks. Cefoxitin and oxacillin disk diffusion results were used as surrogate markers to infer resistance to β-lactam antibiotics in *Staphylococcus* spp., in accordance with CLSI guidelines. Susceptibility interpretation followed CLSI (17) guidelines for all antimicrobials, except for levofloxacin, which was interpreted according to CLSI (18), and fusidic acid and amikacin, which were interpreted according to the European Committee on Antimicrobial Susceptibility Testing (19).

*S. aureus* ATCC^®^ 25922™ was used as the quality control strain for the disk diffusion assays. Multidrug resistance was defined as resistance to three or more classes of antimicrobial agents (20).

### 2.5 Detection of antimicrobial resistance genes

All isolates were screened by PCR for the presence of AMR genes associated with different antibiotic classes. The aminoglycoside resistance genes *aadD* (21), *aph(3*′*)-IIIa* (22), and *aacA-aphD* (23) were investigated, as well as chloramphenicol- and florfenicol-resistance genes *catpC221* (24) and *fexA* (25). The presence of fusidic acid resistance genes *fusB* and *fusC* (26) was also evaluated. Tetracycline-resistance determinants *tet*(M) (27), *tet(L)* (28), and *tet*(K) (29) were screened as well as β-lactam resistance genes *mecA* and *mecC* (30, 31) and the penicillinase gene *blaZ* (24).

Genes conferring resistance to macrolide–lincosamide–streptogramin B (MLS_B_) antibiotics, including *erm*(A) (32), *erm*(B) and *erm*(C) (33), and the efflux-associated gene *vgaC* (34), were also investigated. Trimethoprim-resistance genes *dfrA(S1)* (35) and *dfr(G)* (36) were also screened. Finally, mutations in the quinolone resistance-determining regions of *grlA* and *gyrA* were assessed (28).

All targets were analyzed by PCR amplification. Each reaction contained Supreme NZYTaq II 2x Green Master Mix (NZYTech, Portugal), 0.5 μM of each primer, and template DNA, and amplification was performed using a Biometra Uno II thermal cycler (Analytik Jena, Germany). Negative controls and previously sequenced positive controls were included in every run. PCR products were analyzed by 1.5% (w/v) agarose gel electrophoresis, stained with GreenSafe Premium (NZYTech, Portugal), and visualized under UV light using a UView™ Mini Transilluminator (Bio-Rad, France). Selected PCR products were confirmed by Sanger sequencing to validate the presence of the targeted genes and mutations.

### 2.6 Whole-genome sequencing and bioinformatics analysis

A subset of methicillin-resistant isolates, including *S. pseudintermedius* (*n* = 10) and *Staphylococcus haemolyticus* (*n* = 1), were further characterized by WGS.

Genomic DNA was extracted using the Monarch^®^ HMW DNA Extraction Kit for Tissue (New England Biolabs, US). Oxford Nanopore Technologies (ONT) libraries were prepared with the Rapid Barcoding Sequencing Kit (SQK-RBK114-24, ONT, Oxford, UK) and sequenced on a MinION Mk1B device using a FLO-MIN114 (R10.4.1) flow cell (ONT, Oxford, UK).

Genome assembly and polishing were performed using the EPI2ME (v5.2.5) wf-bacterial-genomes pipeline (v1.4.2). Assembly quality was evaluated with SeqKit v0.8.1 (37). The assemblies presented an average L50 of 11 (ranging from 1 to 122), N50 of 2.38 × 106 (ranging from 6.27 × 103 to 2.80 × 106), and an average sequencing depth of 111 × . Full WGS statistics are provided in Supplementary Table S1. Genome annotation was carried out using the RAST Server (38).

The presence of antimicrobial resistance determinants was assessed using ResFinder 4.1 (39) and the Solu Genomics platform (40). Plasmid replicons were identified with PlasmidFinder (41), and sequence types (STs) were assigned using PubMLST (42). SCC*mec*Finder 1.2 (CGE) (43) was employed to determine the SCC*mec* type of each genome. The presence of virulence factors was evaluated using the Virulence Factor Database (VFDB) (44), putative matches were subsequently manually validated by protein-level alignment against reference sequences.

#### 2.6.1 Phylogenetic analysis

Clonal relatedness among the sequenced *S. pseudintermedius* strains was assessed through a single-nucleotide polymorphism (SNP)-based mapping approach. Draft genomes were aligned against the reference strain *S*. *pseudintermedius* ED99 (GenBank accession: CP002478), and a maximum-likelihood SNP tree was generated using CSI phylogeny 1.4 (45) with default parameters. The resulting phylogeny, together with associated antimicrobial resistance and virulence data, was visualized and explored using the Microreact platform (46).

## 3 Results

### 3.1 Bacterial isolates and host characteristics

A total of 76 bacterial isolates with *Staphylococcus* spp. morphology causing community-acquired otitis externa was obtained from 72 companion animals (54 dogs and 18 cats). The animals' ages ranged from 0.25 to 17 years, with a median age of 6.9 years (*n* = 72), and 44.4% were males (*n* = 32/72).

The most frequent dog breeds in this study were mixed-breed dogs (40.7%, *n* = 22/54), French Bulldogs (29.6%, *n* = 16/54), and Labrador Retrievers (14.8%, *n* = 8/54). All cats included in the study were domestic shorthairs.

Regarding sex, regardless of species, males were more prevalent (59.7%, *n* = 43/72) than females (40.3%, *n* = 29/72). Specifically, male dogs were the most frequent (70.4%, *n* = 38/54), whereas among cats, males were less frequent (27.8%, *n* = 5/18).

The frequency of each staphylococcal species is presented in Table 1. The predominant species was *S. pseudintermedius*, accounting for 45 isolates (59.2%), followed by *S. schleiferi* (*n* = 10, 13.2%). CoNS represented 19 isolates (25.0%) overall, while S. *aureus* was identified in only two cases (2.6%).

**Table 1:** Distribution of *Staphylococcus* species isolated from dogs and cats with otitis externa (*n* = 76) in the Lisbon area, Portugal (2023–2024).

**Staphylococcal species**	**Dog (*n =* 56)**	**Cat (*n =* 20)**	**Total frequency**
	***n*** **(%)**	***n*** **(%)**	***n*** **(%)**
*Staphylococcus pseudintermedius*	42 (75.0)	3 (15.8)	45 (59.2)
*Staphylococcus felis*	0 (0.0)	10 (52.6)	10 (13.2)
*Staphylococcus schleiferi*	9 (16.1)	1 (5.3)	10 (13.2)
*Staphylococcus epidermidis*	0 (0.0)	3 (15.8)	3 (3.9)
*Staphylococcus aureus*	0 (0.0)	2 (10.5)	2 (2.6)
*Staphylococcus haemolyticus*	1 (1.8)	0 (0.0)	1 (1.3)
*Staphylococcus hominis*	1 (1.8)	0 (0.0)	1 (1.3)
*Staphylococcus lugdunensis*	1 (1.8)	0 (0.0)	1 (1.3)
*Staphylococcus pettenkoferi*	1 (1.8)	0 (0.0)	1 (1.3)
*Staphylococcus simulans*	1 (1.8)	0 (0.0)	1 (1.3)
*Staphylococcus succinus*	0 (0.0)	1 (5.3)	1 (1.3)

*n*, number of isolates; %: percentage of the total isolates for each animal species.

### 3.2 Antimicrobial resistance

The frequencies of antimicrobial susceptibility are presented in Table 2 and the distribution of resistance genes in Table 3. Detailed antimicrobial resistance phenotypes and corresponding genes for each strain are displayed in Supplementary Table S2.

**Table 2:** Antimicrobial susceptibility of *Staphylococcus* spp. strains (*n* = 76) recovered from dogs and cats with otitis externa in the Lisbon area, Portugal (2023–2024).

**Antimicrobial class**	**Antimicrobial**	**Susceptible, *n* (%)**	**Intermediate *n* (%)**	**Resistant, *n* (%)**
Aminoglycosides	Amikacin	67 (88.2)	5 (6.6)	4 (5.3)
	Gentamicin	62 (81.6)	1 (1.3)	13 (17.1)
	Tobramycin	61 (80.3)	2 (2.6)	13 (17.1)
β-lactams	Amoxicillin/clavulanate	64 (84.2)	0 (0)	12 (15.8)
	Ampicillin	25 (32.9)	1 (1.3)	50 (65.8)
	Cephalothin	64 (84.2)	0 (0)	12 (15.8)
	Cefovecin	63 (82.9)	1 (1.3)	12 (15.8)
	Cefoxitin	64 (84.2)	0 (0)	12 (15.8)
	Penicillin G	25 (32.9)	1 (1.3)	50 (65.8)
	Oxacillin	64 (84.2)	0 (0)	12 (15.8)
Fluoroquinolones	Enrofloxacin	53 (69.7)	7 (9.2)	16 (21.1)
	Levofloxacin	60 (78.9)	1 (1.3)	15 (19.7)
Macrolides	Erythromycin	46 (60.5)	8 (10.5)	22 (28.9)
Lincosamides	Clindamycin	54 (71.1)	3 (3.9)	19 (25.0)
Phenicols	Chloramphenicol	62 (81.6)	4 (5.3)	10 (13.2)
	Florfenicol	74 (97.4)	1 (1.3)	1 (1.3)
Rifamycins	Rifampicin	73 (96.1)	0 (0)	3 (3.9)
Sulfonamides	Trimethoprim/sulfamethoxazole	57 (75.0)	2 (2.6)	17 (22.4)
Tetracyclines	Doxycycline	41 (53.9)	4 (5.3)	31 (40.8)
	Minocycline	57 (75.0)	14 (18.4)	5 (6.6)
Fusidane derivatives	Fusidic acid	62 (81.6)	0 (0)	14 (18.4)

*n*, number of isolates; %: percentage of the total isolates. Susceptibility was interpreted using the criteria of Clinical and Laboratory Standards Institute (CLSI) (2020) for amoxicillin/clavulanate, ampicillin, cefovecin, cefoxitin, cephalothin, chloramphenicol, clindamycin, doxycycline, enrofloxacin, erythromycin, florfenicol, gentamicin, minocycline, penicillin G, oxacillin, rifampicin, tobramycin, and trimethoprim–sulfamethoxazole; CLSI (19) for levofloxacin; and European Committee on Antimicrobial Susceptibility Testing (19) for fusidic acid and amikacin.

**Table 3:** Frequency of antimicrobial resistance genes detected among *Staphylococcus* spp. strains (*n* = 76) recovered from dogs and cats with otitis externa in the Lisbon area, Portugal (2023–2024).

**Resistance gene**	**Dog (*n =* 56)**	**Cat (*n =* 20)**	**Total frequency**
	***n*** **(%)**	***n*** **(%)**	***n*** **(%)**
*aacA*	9 (16.1)	4 (20.0)	13 (17.1)
*aadD*	0 (0)	1 (5.0)	1 (1.3)
*aph3*	8 (14.3)	3 (15.0)	11 (14.5)
*blaZ*	41 (73.2)	9 (45.0)	50 (65.8)
*cat*	6 (10.7)	1 (5.0)	7 (9.2)
*dfrG*	11 (19.6)	3 (15.0)	14 (18.4)
*dfrA*	0 (0)	0 (0)	0 (0)
*ermA*	0 (0)	0 (0)	0 (0)
*ermB*	11 (19.6)	4 (20.0)	15 (19.7)
*ermC*	0 (0)	0 (0)	0 (0)
*fexA*	1 (1.8)	0 (0)	1 (1.3)
*fusB*	1 (1.8)	1 (5.0)	2 (2.6)
*fusC*	0 (0)	0 (0)	0 (0)
*mecA*	9 (16.1)	3 (15.0)	12 (15.8)
*mecC*	0 (0)	0 (0)	0 (0)
*tet*(M)	21 (37.5)	4 (20.0)	25 (32.9)
*tet*(K)	2 (3.6)	2 (10.0)	4 (5.3)
*tet*(L)	0 (0)	0 (0)	0 (0)
*vgaC*	0 (0)	0 (0)	0 (0)

*n*, number of isolates; %: percentage of isolates within each animal group (dogs and cats).

Most strains were susceptible to the tested antimicrobials, particularly florfenicol (*n* = 74/76, 97.4%), rifampicin (*n* = 73/76, 96.1%), and the aminoglycosides amikacin (*n* = 67/76, 88.2%), gentamicin (*n* = 62/76, 81.6%), and tobramycin (*n* = 61/76, 80.3%). Resistance was most frequent to penicillin G and ampicillin (*n* = 50/76, 65.8%), whereas resistance to other β-lactam agents, including amoxicillin/clavulanate, cephalothin, cefovecin, cefoxitin, and oxacillin, was detected in 12 (15.8%) isolates, all exhibited an MDR profile. All penicillin G-resistant isolates carried the *blaZ* gene. The *mecA* gene was identified in all cefoxitin and oxacillin-resistant staphylococcal isolates (*n* = 12/76, 15.8%), which included 10 *S. pseudintermedius*, one *S. haemolyticus*, and one *Staphylococcus epidermidis* strain. None of the strains carried the *mecC* gene.

Doxycycline resistance was the second most frequent (*n* = 31/76, 40.8%), while resistance to minocycline, another tetracycline agent, remained comparatively low (*n* = 5/76, 6.6%). All strains resistant or intermediate to minocycline were also resistant to doxycycline. Among the 31 doxycycline-resistant strains, 26 (83.9%) carried at least one tetracycline resistance determinant (*tetM* or *tetK*). The *tetK* gene was detected in four isolates, three of which also harbored *tetM*. All minocycline-resistant strains (*n* = 5) carried *tetM* gene, and 12 of 14 strains showing intermediate susceptibility to minocycline also possessed this gene.

Resistance to MLS_B_ (macrolide–lincosamide–streptogramin B) antibiotics was the third most common, with high resistance rates to erythromycin and clindamycin (Table 2). The associated resistance gene *ermB*, was likewise among the most prevalent (*n* = 15/76, 19.7%) (Table 3). Resistance to trimethoprim/sulfamethoxazole was observed in 17 strains (22.4%), of which 14 harbored the *dfrG* gene. Overall, 30 isolates (39.5%) were classified as MDR. Other genes, including *aacA, aph3, aadD, cat, fexA, fusB* and *tet*(K), occur at low frequencies (Table 3).

Resistance to fluoroquinolones was observed in 16 out of the 76 staphylococcal strains (21.1%) when tested against enrofloxacin, and in 15 strains (19.7%) against levofloxacin. Sequencing of the Quinolone Resistance-Determining Regions of *grlA* and *gyrA* revealed the presence of characteristic point mutations associated with resistance (Supplementary Table S2). The most frequent mutation pattern, GrlA(S80I)/GyrA(S84L), was detected in ten *S. pseudintermedius* strains and two *S. schleiferi* strains and was consistently associated with a resistant phenotype to both enrofloxacin and levofloxacin. The GrlA(S80R) substitution, alone or in combination with GyrA(S84L), was identified in two trains, both showing resistance to the two fluoroquinolones tested. One *S. pseudintermedius* strain (S7) harbored a single GyrA(S84L) mutation and was resistant to both drugs, while another strain (O42) carried only GrlA(S80I) and exhibited resistance to enrofloxacin but remained susceptible to levofloxacin. Interestingly, two strains (*S. felis* O11 and *S. pseudintermedius* O57) lacked mutations in *grlA* and *gyrA* but still displayed phenotypic resistance to at least one fluoroquinolone, suggesting the possible involvement of alternative resistance mechanisms such as efflux pump overexpression or permeability changes.

### 3.3 Genomic analysis

**Figure 1:**
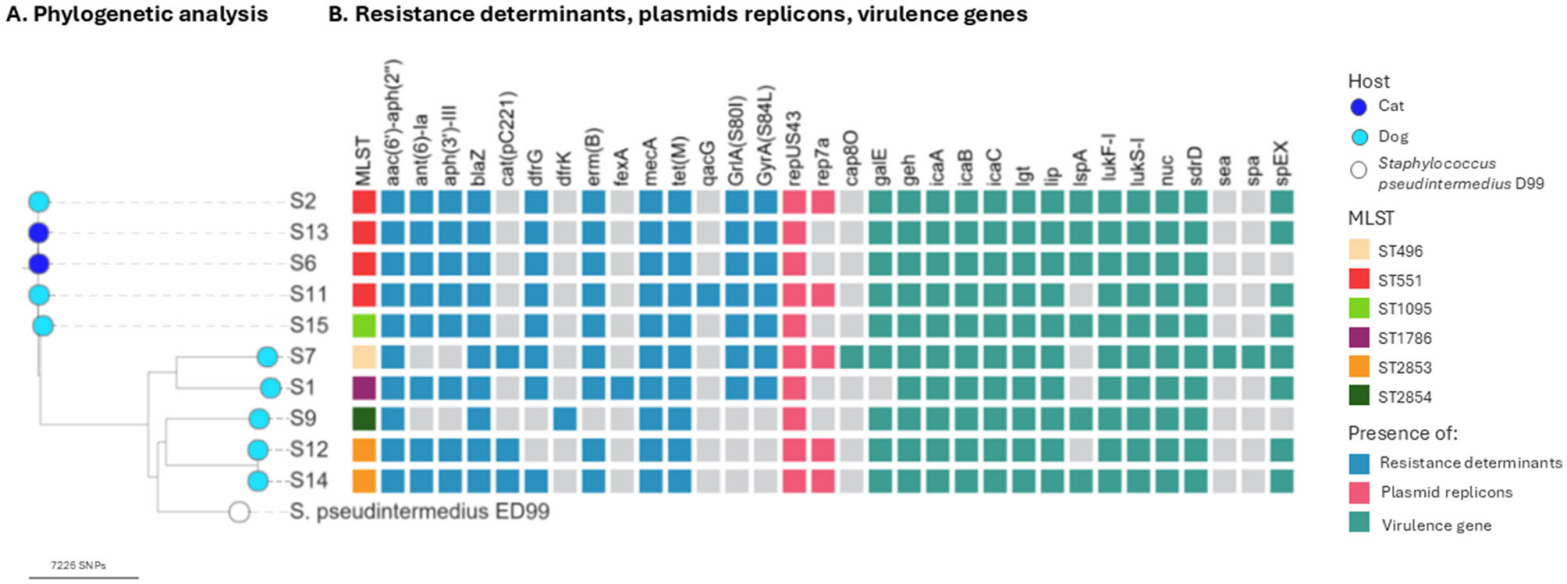
Core genome SNP analysis and genetic features of methicillin-resistant *Staphylococcus pseudintermedius* strains causing otitis externa in companion animals from the Lisbon area, Portugal, 2023–2024. **(A)** Phylogenetic analysis of the 10 sequenced methicillin-resistant *S. pseudintermedius* strains and the *S. pseudintermedius* D99 strains. The SNP tree was created with CSI phylogeny. **(B)** Heatmap shows the sequence types, antimicrobial resistance determinants, plasmid replicons, and virulence factors for each strain (see color key right side of the figure).

WGS was performed on the 10 *mecA*-positive *S. pseudintermedius* isolates and the *S. haemolyticus* strain to further characterize their genomic features. Among the *S. pseudintermedius* isolates, four belonged to the prevalent European lineage ST551, whereas two were assigned to a novel sequence type ST2853 and another to novel sequence type ST2854. The remaining strains corresponded to ST1786, ST496, and ST1095. The *S. haemolyticus* strain belonged to ST56 (Figure 1; Supplementary Table S3).

Core SNP-based phylogenetic analysis revealed that the two *S. pseudintermedius* isolates assigned to ST2853 (S12 and S14) differed by only two single-nucleotide polymorphisms (SNPs), suggesting a close genetic relationship. These strains were obtained from two dogs with otitis, from different households (Figure 1).

*In silico* analysis identified 12 antimicrobial resistance genes, confirming the presence of previously detected resistance determinants [*blaZ, mecA, tet*(M)*, erm*(B)*, dfrG, aacA, aph3, cat, fexA, fusB, tet*(K)], as well as the *qacG* gene, associated with resistance to quaternary ammonium compounds. Mutations in *gyrA* and *grlA*, conferring fluoroquinolone resistance, were also detected.

Plasmid analysis revealed that all MRSP strains carried plasmid replicons, with repUS43 being the most prevalent, detected in eight strains, either alone or in combination with rep7a (Figure 1; Supplementary Table S3). Notably, the methicillin-resistant *S. haemolyticus* strain did not harbor any identifiable plasmid replicons.

Regarding SCC*mec* characterization, type Vc (5C2&5) was the most prevalent cassette, detected in seven *S. pseudintermedius* strains and in the methicillin-resistant *S. haemolyticus* strain S4. Clonal strains S12 and S14 carried SCC*mec* type IVc (2B), whereas the cassette type could not be determined for strain S9.

### 3.4 Virulence determinants

In this study, the analysis of virulence determinants was performed only on the 11 methicillin-resistant isolates subjected to WGS. A total of 18 virulence factors genes were identified across the sequenced staphylococcal genomes, encompassing surface adhesion proteins, toxins, and biofilm-associated factors (Figure 1; Supplementary Table S3). All MRSP strains carried the *lukF-I* and *lukS-I* genes encoding the bicomponent leukotoxin Luk-I, as well as the *nuc* gene, the *icaA, icaB* and *icaC* genes, linked to biofilm formation, and the *lgt* gene, which mediates lipoprotein lipidation. Two YSIRK-domain-containing triacylglycerol lipase genes (*geh* and *lip*), involved in lipid hydrolysis and tissue invasion, were also present in all MRSP genomes.

All MRSP genomes carried the *sdrD* gene encoding a serine–aspartate repeat surface protein, and seven isolates harbored the *spEX* gene, encoding a staphylococcal exotoxin. The *lspA* gene, encoding lipoprotein signal peptidase, was detected in six MSRP strains. One strain (S7) carried both the *spa* and *sea* genes, which encode staphylococcal protein A and enterotoxin A (SEA), respectively—two important virulence factors implicated in immune evasion and toxin-mediated pathology.

The methicillin-resistant *S. haemolyticus* strain (S4) was also carried the virulence genes *lip* along with *atl*, and *ebpS* gene that encodes an elastin-binding surface protein that mediates attachment to host connective tissues. In addition, this strain harbored the resistance genes *blaZ, fusB, mecA, mph(C)*, and *msr(A)* (Supplementary Table S3); the latter two genes encode a macrolide phosphotransferase and an efflux pump associated with macrolide and streptogramin B resistance, respectively.

## 4 Discussion

*Staphylococcus* spp., particularly *S. pseudintermedius* and *S. aureus*, are among the main bacterial agents responsible for skin, ear, and soft tissue infections in companion animals (7). The emergence and dissemination of AMR within these species represent a growing public health concern (4). Although zoonotic transmission of *S. pseudintermedius* from animals to humans is considered rare, the potential for bidirectional transmission between pets and humans supports the relevance of these pathogens at the animal–human interface (4). Consequently, understanding the epidemiology and resistance mechanisms of staphylococci isolated from companion animals is crucial to promote prudent antimicrobial use and to inform effective infection control strategies (47).

In this study, *S. pseudintermedius* was the most predominant species, representing 59.2% of all isolates from ear swabs. This finding aligns with previous epidemiological data showing the predominance of *S. pseudintermedius* among staphylococcal infections in companion animals. In a 16-year surveillance study in Portugal, this species accounted for 70.6% (*n* = 446/632) of isolates from companion animals (6). Likewise, a study conducted in Northern Portugal reported *S. pseudintermedius* as the most common species in dogs (62%) and cats (30%) with clinical infections (48), while a recent Romanian investigation identified it in 40% of canine otitis externa cases (9).

Consistent with previous reports, resistance to tetracyclines was among the highest observed in this study. Doxycycline resistance reached 40.8%, a frequency comparable to findings from companion animals with otitis externa staphylococcal isolates in Romania (37.8%) (9) and Spain (41.7%) (49). Tetracyclines are widely used in veterinary medicine due to their broad-spectrum activity, accessibility, and low cost (50), factors that likely contribute to the sustained selective pressure and persistence of resistant clones across clinical settings.

An important finding is that 39.5% of the strains in this study were classified as MDR, including those exhibiting resistance to cefoxitin and/or oxacillin and classified as MRS (15.8%). The frequency of MRS detected here is in line with reports from other European countries (6, 48, 89), although lower than those reported in dermatological samples from dogs in the United States (51). Such discrepancies likely reflect regional differences in antimicrobial usage practices and resistance ecology, as highlighted in other comparative studies (50, 52, 53). Importantly, methicillin resistance was consistently associated with the presence of *mecA*, supporting evidence that this remains the dominant determinant of β-lactam resistance in staphylococci circulating in companion animals (7, 51, 54). Given that β-lactams also represent the most commonly prescribed antimicrobial class in human medicine (50), the presence of MRS in companion animals reinforces the relevance of a One Health approach, as these strains may disseminate across host barriers and compromise therapeutic options.

The prevailing SCC*mec* type identified among the *mecA*-positive strains was type V—contrasting with other European studies where SCC*mec* type III has been reported as the predominant (55–57). This observation further illustrates the geographical variability in SCC*mec* distribution and may signal ongoing microevolution within local MRSP lineages.

As expected, resistance to florfenicol was rare, with only one isolate carrying the *fexA* gene. This aligns with the recommended use of florfenicol as a second-line agent for the management of MRSP and ESBL-producing *Escherichia coli* in dogs (58), which may limit selective pressure and help preserve its clinical efficacy.

WGS analysis provided further insight into the epidemiology of the MRS strains. Most MRSP strains belonged to the internationally disseminated ST551 lineage (*n* = 4), a lineage repeatedly reported across Europe and increasingly recognized in companion animals (57, 59–61), environmental samples from veterinary clinics (62), and even sporadically in humans (63). In addition, other established MRSP lineages were detected, including ST496, which has previously been associated with extensive resistance to veterinary-licensed antimicrobials (64). Four isolates corresponded to novel sequence types (ST2853–ST2855), reflecting ongoing diversification in the MRSP population.

Two strains of the novel sequence type ST2853 (S12 and S14), differed by only two SNPs, strongly suggesting a shared recent origin. Although both animals had visited the same veterinary hospital, they originated from different owners, and the infections were already present upon admission. Therefore, these findings are consistent with a community-acquired transmission rather than healthcare-associated transmission. This likely reflects the circulation of this lineage in the region and highlights the need for further studies to better characterize its distribution and epidemiology.

While veterinary hospitals are recognized in the literature as hotspots for the selection and dissemination of antimicrobial-resistant staphylococci (65, 66), *S. pseudintermedius* can persist on surfaces for up to 10 weeks (67), meaning that environmental reservoirs in the wider community could contribute to its transmission. Targeted investigations—including environmental sampling and screening of in-contact animals—would help clarify the dynamics of these transmission pathways (68).

Building on the genomic background, the virulence gene content of these MRS strains further illustrates their pathogenic and persistence potential. Genes encoding the bicomponent leukotoxin Luk-I were consistently detected across the MRSP strains. Leukocidins target host defense cells and erythrocytes, facilitating survival and spread within host tissues (69, 70). The widespread presence of Luk-I among MRSP has been documented in multiple studies and is regarded as a hallmark virulence determinant in this bacterial species, supporting its role in host adaptation and pathogenic potential (69). Adhesion and biofilm-associated determinants were consistently identified among the MRSP genomes. The carried genes belonging to the *icaBDCA* operon, which mediates PIA-dependent biofilm matrix synthesis, were present in all strains (71). Likewise, *sdrD*, encoding a serine–aspartate repeat protein that promotes adhesion, persistence and evasion from neutrophil-mediated killing (2, 11), was ubiquitous. The *nuc* gene, which encodes a thermostable nuclease involved in extracellular DNA degradation and biofilm dispersal, was also detected in all MRSP genomes (72). Although the *galE* gene was also present, its role in MRSP biology remains unclear. In other bacterial species, GalE (UDP-galactose-4-epimerase) contributes to biofilm formation, cell-surface architecture and virulence (73), but analogous functions have not been demonstrated in *S. pseudintermedius*. Functional studies will therefore be required to determine whether *galE* influences biofilm physiology or host interaction in this species. Notably, Pompilio et al. (10) reported that *S. pseudintermedius* strains associated with human wound infections can form ultrastructurally complex biofilms, which provide a protective environment against antibiotics and enhance pathogenic potential (10).

Lgt, identified in all MRSP genomes, catalyzes the first committed step in bacterial lipoprotein maturation. In *S. aureus*, disruption of *lgt* results in impaired growth under nutrient-limiting conditions, and a markedly reduced ability to stimulate host proinflammatory cytokine production (74, 75). Its detection here suggests that MRSP strains retain the molecular machinery needed for lipoprotein processing and host interaction, although functional studies are still needed in *S. pseudintermedius*. A second lipoprotein-processing gene, *lspA*, was identified in six isolates belonging to ST551, ST1095, ST2853, and ST2854. LspA removes signal peptides from prolipoproteins after Lgt-mediated diacylglycerol modification. Disruption of *lspA* in staphylococci alters cell envelope stability (76, 77). Although not universally distributed among MRSP in this dataset, its presence in several distinct lineages indicates that lipoprotein maturation pathways may vary across clones.

Two YSIRK-domain triacylglycerol lipases—Geh and Lip—were identified in all MRSP. These enzymes promote hydrolysis of host lipids and contribute to tissue invasion (78). Their secretion via a YSIRK-G/S signal peptide likely facilitates surface localization and interaction with host environments.

The *spEX* gene, encoding a staphylococcal exotoxin-like protein of the SET/SSL family, was present in nearly all MRSP except ST2854. SpEX interferes with neutrophil function and enhances colonization, and has been found in isolates from both human and canine infections, including documented zoonotic transmission events (79).

Among all MRSP strains, only one (S7-ST496) carried the *spa* gene, encoding staphylococcal protein A, a well-characterized virulence determinant widely studied in *S. aureus* and frequently detected in strains from companion animals (6, 80). Its sporadic presence in *S. pseudintermedius* aligns with previous evidence that *spa* is less prevalent and may be subject to lineage-specific acquisition or loss. The same isolate also harbored *sea*, a mobile element–associated superantigen gene rarely identified in *S. pseudintermedius*, suggesting potential horizontal transfer from *S. aureus* (1).

The methicillin-resistant *S. haemolyticus* isolate belonged to ST56, a lineage previously detected on isolates from blood cultures collected in humans from Nigeria and India associated with multidrug-resistant phenotypes (81, 82). Its detection in a companion animal context reinforces growing concerns regarding the ecological flexibility and cross-host dissemination of staphylococci isolates. This strain carried *atl, ebpS* and *lip* virulence genes. The *atl* gene encodes a bifunctional autolysin involved in cell separation, adhesion and biofilm development (83), while *ebpS* mediates elastin binding—enhancing attachment to host tissues (84). Together, these virulence factors demonstrate the potential of ST56 *S. haemolyticus* to persist in host tissues and withstand antimicrobial pressure, underscoring the clinical importance of monitoring these species within companion-animal populations.

Virulence traits identified here—including biofilm-associated genes, adhesion factors, and immune-evasion mechanisms— support the zoonotic potential of MRSP. The ability of these strains to colonize human skin and cause infections has been documented (84). Although direct evidence on the added risk of specific pet-owner behaviors is limited, prudent practices such as avoiding face licking, hand-to-mouth contact, and sharing beds may help reduce potential transmission (85, 86).

The identification of repUS43 and rep7a plasmid replicons in MRSP strains aligns with reports of these plasmids in other Gram-positive bacteria from human sources (87, 88). These findings suggest the potential role of mobile genetic elements in the dissemination of resistance and virulence determinants across bacterial populations. They underscore the importance of integrating plasmid surveillance into a One Health perspective to better understand the dynamics of antimicrobial resistance across human and animal populations.

Although this study provides valuable insights, some limitations should be considered. The sample size was relatively small, and the diversity of dog breeds and the limited number of cats prevented analysis of potential associations between host factors (such as breed, age, or sex) and *Staphylococcus* spp. prevalence or resistance patterns. Nonetheless, inclusion of feline isolates remains particularly valuable, as few studies have characterized *Staphylococcus* spp. from cats with otitis externa.

This study sheds light on antimicrobial resistance patterns and the prevalence of multidrug-resistant methicillin-resistant staphylococci in companion animals. The findings emphasize the zoonotic relevance of these pathogens and support practical measures for control, including routine antimicrobial susceptibility testing, reduced reliance on empirical therapies, strict hygiene and infection control protocols in clinics and households, and ongoing surveillance of resistant strains. Implementing these strategies can help mitigate antimicrobial misuse and limit the spread of methicillin-resistant staphylococci, protecting both animal and human health.

## 5 Conclusion

*Staphylococcus* spp. plays an important clinical and epidemiological role in otitis externa of companion animals. The results demonstrate a notable prevalence of antimicrobial resistance, including methicillin-resistant strains, and highlight the potential zoonotic relevance of these bacteria. The findings support the need for ongoing surveillance, effective infection control measures, and evidence-based antimicrobial stewardship in veterinary practice to limit the spread of multidrug-resistant staphylococci at the animal–human interface.

## Data Availability

The bacterial sequences generated in this study were deposited in the NCBI GenBank database under BioProject accession number PRJNA1303419. The individual GenBank accession numbers for the 11 isolates are: SAMN50513626, SAMN50514037, SAMN50518053, SAMN50519026, SAMN50519455, SAMN50520279, SAMN50554468, SAMN50554649, SAMN50554641, SAMN50554669, SAMN50554651.
