# Supplementary material for: Genomic insights into multidrug resistance and virulence of methicillin-resistant Staphylococcus pseudintermedius from companion animal otitis
Source: Front Vet Sci. 2026 Jan 28;13:1759838. doi: 10.3389/fvets.2026.1759838 (PMC12890695; doi:10.3389/fvets.2026.1759838)
Supplement: Supplementary file 1 [file Data_Sheet_1.pdf]

Supplementary Table S1. Overview of assembly statistics

Strain code	%GC	Total length	Deph	No. Contigs	N50	L50
S1	37.28	2879887	48x	5	2.80E+06	1
S2	37.42	2715029	112x	1	2.72E+06	1
S4	33.00	2510869	15x	484	6.27E+03	122
S6	37.41	2715791	38x	1	2.72E+06	1
S7	37.47	2747564	50x	8	1.69E+06	1
S9	37.56	2657028	40x	1	2.66E+06	1
S10	37.41	2715767	21x	1	2.72E+06	1
S11	37.41	2713538	575x	2	2.71E+06	1
S12	37.35	2716995	106x	4	2.58E+06	1
S13	37.47	2664650	118x	1	2.66E+06	1
S14	37.46	2676110	108x	6	2.59E+06	1
S15	37.43	2759632	109x	1	2.76E+06	1

Supplementary Table S2. Characterization of *Staphylococcus* isolates recovered from dogs and cats with otitis externa.

Strain code	Host	Bacterial species	Resistance phenotype	Resistance determinats	GrlA and GyrA Mutations
O1	Dog	*Staphylococcus pseudintermedius*	AK, AMP, CN, DA, DO, E, ENR, FD, LEV, P, RD, SXT, TOB	*aacA, aph3, blaZ, dfrG, erm*(B), *tet(K)*	GrlA(S80I), GyrA(S84L)
S15	Dog	*Staphylococcus pseudintermedius*	AMC, AMP, CN, CVN, DA, DO, E, ENR, FOX, KF, LEV, OX, P, SXT	*aacA, aph3, blaZ, dfrG, erm*(B), *mecA, tet*(M)	GrlA(S80I), GyrA(S84L)
S1	Dog	*Staphylococcus pseudintermedius*	AK, AMP, AMC, C, CN, CVN, DA, DO, E, ENR, FFC, FOX, KF, LEV, OX, P, SXT, TOB	*aacA, aph3, blaZ, dfrG, erm*(B), *fexA, mecA, tet*(M)	GrlA(S80I), GyrA(S84L)
O2	Dog	*Staphylococcus pseudintermedius*	AMP, CN, DA, DO, E, P, TOB	*blaZ*	None
O3	Cat	*Staphylococcus epidermidis*	AK, DA, DO, E, FD, P	*fusB*	None
O4	Cat	*Staphylococcus felis*	None	None	None
O5	Dog	*Staphylococcus schleiferi*	None	None	None
O6	Dog	*Staphylococcus pseudintermedius*	AMP,P	*blaZ*	None
O7	Dog	*Staphylococcus pseudintermedius*	None	None	None
O8	Cat	*Staphylococcus epidermidis*	AMP, FD, P	*blaZ*	None
O9	Cat	*Staphylococcus felis*	None	None	None
O10	Dog	*Staphylococcus pseudintermedius*	AMP, P	*blaZ*	None
O11	Cat	*Staphylococcus felis*	CN, ENR, LEV, P, TOB	None	None
S2	Dog	*Staphylococcus pseudintermedius*	AMC, AMP, CN, CVN, DA, DO, E, ENR, FOX, KF, LEV, OX, P, SXT, TOB	*aacA, aph3, blaZ, dfrG, erm*(B), *mecA, tet*(M), *tet*(K)	GrlA(S80I), GyrA(S84L)
O12	Dog	*Staphylococcus pseudintermedius*	AMP, DO, FD, P	*blaZ, tet(M)*	None
O13	Dog	*Staphylococcus pseudintermedius*	None	None	None
S3	Dog	*Staphylococcus pseudintermedius*	AMP, C, DA, E, P	*blaZ, cat, erm(B)*	None

Strain code	Host	Bacterial species	Resistance phenotype	Resistance determinats	GrlA and GyrA Mutations
O14	Dog	*Staphylococcus lugdunensis*	None	None	None
O15	Dog	*Staphylococcus pseudintermedius*	AMP, DO, P	*blaZ, tet(M)*	None
O16	Cat	*Staphylococcus felis*	None	None	None
O17	Dog	*Staphylococcus pseudintermedius*	AMP, DO, P	*blaZ, tet(M)*	None
O18	Dog	*Staphylococcus pseudintermedius*	None	None	None
O19	Dog	*Staphylococcus pseudintermedius*	AMP, DO, P	*blaZ, tet(M)*	None
O20	Dog	*Staphylococcus pseudintermedius*	AMP, P	*blaZ*	None
O21	Dog	*Staphylococcus schleiferi*	FD	None	None
O22	Dog	*Staphylococcus pseudintermedius*	AMP, P	*blaZ*	None
S4	Dog	*Staphylococcus heamolyticus*	AMC, AMP, CNV, E, FD, FOX, KF, OX, P	*blaZ, fusB, mecA*	None
S5	Cat	*Staphylococcus epidermidis*	AMC, AMP, C, CVN, DA, FOX, KF, OX, P, TOB	*aacA, aadD, blaZ, cat, mecA*	None
O25	Dog	*Staphylococcus pseudintermedius*	AMP, C, DA, E, P	*blaZ*	None
O26	Dog	*Staphylococcus schleiferi*	None	None	None
O27	Dog	*Staphylococcus schleiferi*	SXT	*dfrG*	None
O28	Dog	*Staphylococcus schleiferi*	ENR, LEV	None	GrlA(S80I), GyrA(S84L)
S6	Cat	*Staphylococcus pseudintermedius*	AMC, AMP, CN, CVN, DA, DO, E, ENR, FOX, KF, LEV, MH, OX, P, SXT, TOB	*aacA, aph3, blaZ, dfrG, erm*(B), *mecA, tet*(M)	GrlA(S80I), GyrA(S84L)
O29	Dog	*Staphylococcus pseudintermedius*	AMP, C, DA, DO, E, ENR, LEV, MH, P, TOB, SXT,	*aacA, aph3, blaZ, cat, dfrG, erm(B), tet(M)*	GrlA(S80I), GyrA(S84L)
O30	Cat	*Staphylococcus aureus*	AMP, DA, E, P	*blaZ*	None
O31	Dog	*Staphylococcus pseudintermedius*	AMP, DO, FD, P	*blaZ, tet(M)*	None
O32	Dog	*Staphylococcus pseudintermedius*	AMP, P	*blaZ*	None

Strain code	Host	Bacterial species	Resistance phenotype	Resistance determinats	GrlA and GyrA Mutations
O33	Dog	*Staphylococcus pseudintermedius*	AMP, P	*blaZ*	None
O34	Cat	*Staphylococcus felis*	None	None	None
S7	Dog	*Staphylococcus pseudintermedius*	AMC, AMP, C, CN, CVN, DA, DO, E, ENR, FOX, KF, LEV, OX, P, SXT	*aacA, blaZ, cat, dfrG, erm*(B)*, mecA, tet*(M)	GyrA(S84L)
O35	Dog	*Staphylococcus schleiferi*	None	None	None
S9	Dog	*Staphylococcus pseudintermedius*	AMC, AMP, CVN, DO, FOX, KF, OX, P, SXT	*blaZ, mecA, tet*(M)	None
O36	Cat	*Staphylococcus succinus*	AMP, DO	*blaZ, tet*(M)*, tet*(K)	None
S11	Dog	*Staphylococcus pseudintermedius*	AMC, AMP, CN, CVN, DA, DO, E, ENR, FOX, KF, LEV, OX, P, SXT, TOB	*aacA, aph3, blaZ, dfrG, erm*(B)*, mecA, tet*(M)	GrlA(S80I), GyrA(S84L)
O37	Cat	*Staphylococcus felis*	None	None	None
O38	Dog	*Staphylococcus hominis*	None	None	None
S12	Dog	*Staphylococcus pseudintermedius*	AMC, AMP, C, CN, CVN, DA, DO, E, FOX, KF, MH, OX, P, SXT, TOB	*aacA, aph3, blaZ, cat, erm*(B)*, mecA, tet*(M)	None
O39	Dog	*Staphylococcus pseudintermedius*	AK, AMP, C, FD, P	*blaZ*	None
O40	Cat	*Staphylococcus felis*	E	*erm(B)*	None
O41	Dog	*Staphylococcus pseudintermedius*	AMP, DO, ENR, FD, LEV, P	*blaZ*	GrlA(S80R), GyrA(S84L)
O42	Dog	*Staphylococcus pseudintermedius*	DO, ENR	*tet(M)*	GrlA(S80I)
S13	Cat	*Staphylococcus pseudintermedius*	AMC, AMP, CN, CVN, DA, DO, E, ENR, FD, FOX, KF, LEV, OX, P, SXT, TOB	*aacA, aph3, dfrG, blaZ, mecA, tet*(M)	GrlA(S80I), GyrA(S84L)
O43	Dog	*Staphylococcus simulans*	AMP, DO, FD, P	*blaZ*	None
O44	Dog	*Staphylococcus pseudintermedius*	AMP, DO, P, SXT	*blaZ, dfrG, tet(M)*	None
O45	Dog	*Staphylococcus pseudintermedius*	AMP, DO, P, RD	*blaZ, tet(M)*	None
O46	Dog	*Staphylococcus pseudintermedius*	AMP, P	*blaZ*	None
O47	Dog	*Staphylococcus pettenkoferi*	None	None	None

Strain code	Host	Bacterial species	Resistance phenotype	Resistance determinats	GrlA and GyrA Mutations
O48	Dog	*Staphylococcus pseudintermedius*	SXT	*dfrG*	None
O49	Dog	*Staphylococcus pseudintermedius*	AMP, DO, FD, P, SXT	*blaZ*	None
O50	Cat	*Staphylococcus felis*	None	None	None
O51	Dog	*Staphylococcus pseudintermedius*	AMP, DO, P	*blaZ, tet(M)*	None
O52	Dog	*Staphylococcus schleiferi*	AMP, P	*blaZ*	None
O53	Cat	*Staphylococcus aureus*	AMP, P	*blaZ*	None
O54	Dog	*Staphylococcus pseudintermedius*	AMP, P	*blaZ*	None
O55	Cat	*Staphylococcus felis*	None	None	None
O56	Dog	*Staphylococcus pseudintermedius*	AMP, C, DA, E, P	*blaZ, cat, erm(B)*	None
O57	Dog	*Staphylococcus pseudintermedius*	AMP, DO, E, ENR, FD, LEV, P, RD	*blaZ, tet(M)*	None
O58	Dog	*Staphylococcus pseudintermedius*	AMP, DO	*blaZ, tet(M)*	None
O59	Cat	*Staphylococcus felis*	None	None	None
O60	Dog	*Staphylococcus schleiferi*	AMP, ENR, FD, LEV, P	*blaZ*	GrlA(S80R), GyrA(S84L)
O61	Cat	*Staphylococcus schleiferi*	AMP, P	*blaZ*	None
O62	Cat	*Staphylococcus pseudintermedius*	AMP, CN, DO, ENR, LEV, MH, P, TOB	*aacA, aph3, blaZ, dfrG, erm(B), tet(M), tet(K)*	GrlA(S80I), GyrA(S84L)
O63	Dog	*Staphylococcus pseudintermedius*	AMP, DA, E, P	*blaZ*	None
S14	Dog	*Staphylococcus pseudintermedius*	AMC, AMP, C, CN, CVN, DA, DO, E, FOX, KF, MH, OX, TOB	*aacA, aph3, blaZ, cat, dfrG, erm*(B)*, mecA, tet*(M)	None
O64	Dog	*Staphylococcus schleiferi*	AMP, P	*blaZ*	None
O65	Dog	*Staphylococcus pseudintermedius*	None	None	None

Legend: AK, amikacin (30 µg); AMC, amoxicillin/clavulanate (30 µg); AMP, ampicillin (10 µg); C, chloramphenicol (30 µg); CN, gentamicin (10 µg); CVN, cefovecin (30 µg); DA, clindamycin (2 µg); DO, doxycycline (30 µg); E, erythromycin (15 µg); ENR, enrofloxacin (5 µg); FD, fusidic acid (10 µg); FFC, florfenicol (30 µg); FOX, cefoxitin (30 µg); KF, cephalothin (30 µg); LEV, levofloxacin (5 µg); MH, minocycline (30 µg); P, penicillin G (10 U); OX, oxacillin (1 µg); RD, rifampicin (5 µg); SXT, trimethoprim–sulfamethoxazole (25 µg); TOB, tobramycin (10 µg) (CLSI 2020, CLSI 2024, EUCAST 2025).

Supplementary Table S3. Genotypic and phenotypic characteristics of Methicillin resistant *Staphylococcus* spp. strains causing otitis externa in dogs and cats, Portugal 2023-2024.

Strain code	Host	Bacterial species	MLST	Plasmid's replicons	Antimicrobial resistant determinants		SCC*mec* type	Virulence genes
					Acquired resistance genes	Chromosomal point mutations		
S1	Dog	*Staphylococcus pseudintermedius*	1786	repUS43	*aac(6')-aph(2''), ant(6)-Ia, aph(3')-III, blaZ, dfrG, erm*(B), *fexA, mecA, tet*(M)	GrlA(S80I), GyrA(S84L)	Vc (5C2&5)	*geh, icaA, icaB, icaC, lgt, lip, lukF-I, lukS-I, nuc, sasG, spEX*
S2	Dog	*Staphylococcus pseudintermedius*	551	rep7a, repUS43	*aac(6')-aph(2''), ant(6)-Ia, aph(3')-III, blaZ, dfrG, erm*(B), *mecA, tet*(M), *tet*(K)	GrlA(S80I), GyrA(S84L)	Vc (5C2&5)	*galE, geh, icaA, icaB, icaC, lgt, lip, lspA, lukF-I, lukS-I, nuc, sdrD, spEX*
S4	Dog	*Staphylococcus haemolyticus*	56	None	*blaZ, fusB, mecA, mph(C), msr(A)*	None	Vc (5C2&5)	*atl, ebpS, lip, nuc*
S6	Dog	*Staphylococcus pseudintermedius*	551	repUS43	*aac(6')-aph(2''), ant(6)-Ia, aph(3')-III, blaZ, dfrG, erm*(B), *mecA, tet*(M)	GrlA(S80I), GyrA(S84L)	Vc (5C2&5)	*galE, geh, icaA, icaB, icaC, lgt, lip, lspA, lukF-I, lukS-I, nuc, sdrD*
S7	Dog	*Staphylococcus pseudintermedius*	496	rep7a, repUS43	*aac(6')-aph(2''), blaZ, cat(pC221), dfrG, erm(B), mecA, tet*(M)	GrlA(S80I), GyrA(S84L)	V (5C2&5)	*cap8O, galE, geh, icaA, icaB, icaC, lgt, lip, lukF-I, lukS-I, nuc, sdrD, sea, spa, spEX*
S9	Dog	*Staphylococcus pseudintermedius*	2854	repUS43	*blaZ, dfrK, mecA, tet*(M)	None	None	*galE, geh, icaA, icaB, icaC, lgt, lip, lspA, lukF-I, lukS-I, nuc, sdrD*
S11	Dog	*Staphylococcus pseudintermedius*	551	rep7a, repUS43	*aac(6')-aph(2''), ant(6)-Ia, aph(3')-III, blaZ, dfrG, erm*(B), *mecA, tet*(M), *qacG*	GrlA(S80I), GyrA(S84L)	Vc (5C2&5)	*galE, geh, icaA, icaB, icaC, lgt, lip, lukF-I, lukS-I, nuc, sdrD, spEX*

Strain code	Host	Bacterial species	MLST	Plasmid's replicons	Antimicrobial resistant determinants		SCC*mec* type	Virulence genes
					**Acquired resistance genes**	**Chromosomal point mutations**		
S12	Dog	*Staphylococcus pseudintermedius*	2853	rep7a, repUS43	*aac(6')-aph(2''), ant(6)-Ia, aph(3')-III, blaZ, cat(pC221), erm*(B), *mecA, tet*(M)	None	IVc (2B)	*galE, geh, icaA, icaB, icaC, lgt, lip, lukF-I, lukS-I, nuc, sdrD, spEX*
S13	Cat	*Staphylococcus pseudintermedius*	551	repUS43	*aac(6')-aph(2''), ant(6)-Ia, aph(3')-III, blaZ, dfrG, erm*(B), *mecA, tet*(M)	GrlA(S80I), GyrA(S84L)	Vc (5C2&5)	*galE, geh, icaA, icaB, icaC, lgt, lip, lspA, lukF-I, lukS-I, nuc, sdrD, spEX*
S14	Dog	*Staphylococcus pseudintermedius*	2853	rep7a, repUS43	*aac(6')-aph(2''), ant(6)-Ia, aph(3')-III, blaZ, cat(pC221), dfrG, erm*(B), *mecA, tet*(M)	None	IVc (2B)	*galE, geh, icaA, icaB, icaC, lgt, lip, lspA, lukF-I, lukS-I, nuc, sdrD, spEX*
S15	Dog	*Staphylococcus pseudintermedius*	1095	repUS43	*aac(6')-aph(2''), ant(6)-Ia, aph(3')-III, blaZ, dfrG, erm*(B), *mecA, tet*(M)	GrlA(S80I), GyrA(S84L)	Vc (5C2&5)	*galE, geh, icaA, icaB, icaC, lgt, lip, lspA, lukF-I, lukS-I, nuc, sdrD, spEX*

Legend: *atl*, bifunctional autolysin; *cap8O*, type 8 capsular polysaccharide synthesis protein Cap8O; *ebpS*, Elastin binding protein EbpS; *galE*, UDP-galactose-4-epimerase; *geh*, Triacylglycerol lipase EC 3.1.1.3; *icaA*, N-acetylglicosaminatranferase; *icaB*, PIA de-acetylase; *icaC*, intercellular adhesion protein; *lgt*, Prolipoprotein diacylglyceryl transferase; *lip*, YSIRK-targeted triacylglycerol lipase; *lspA*, Lipoprotein signal peptidase; *lukF-I*, leukocidin/hemolysin toxin family protein subunit F; *lukS-I*, leukocidin/hemolysin toxin family protein subunit S; *nuc*, Thermonuclease; *sdrD*, serine-aspartate repeat containing protein D; *sea*, Enterotoxin A; *spa*, staphylococcal protein A; *spEX*, exotoxin SpEX.
